# Addressing the Multisystemic Impacts of Nephropathic Cystinosis in an Adult

**DOI:** 10.1016/j.ekir.2024.10.039

**Published:** 2025-03-04

**Authors:** Jeanine R. Jarnes, Rebekah S. Palmer, Chester B. Whitley

**Affiliations:** 1 University of Minnesota, Department of Pediatrics, Minneapolis, Minnesota, USA; 2 Next Generation of Cystinosis, Ankeny, Iowa, USA

## Introduction

**Figure 1:**
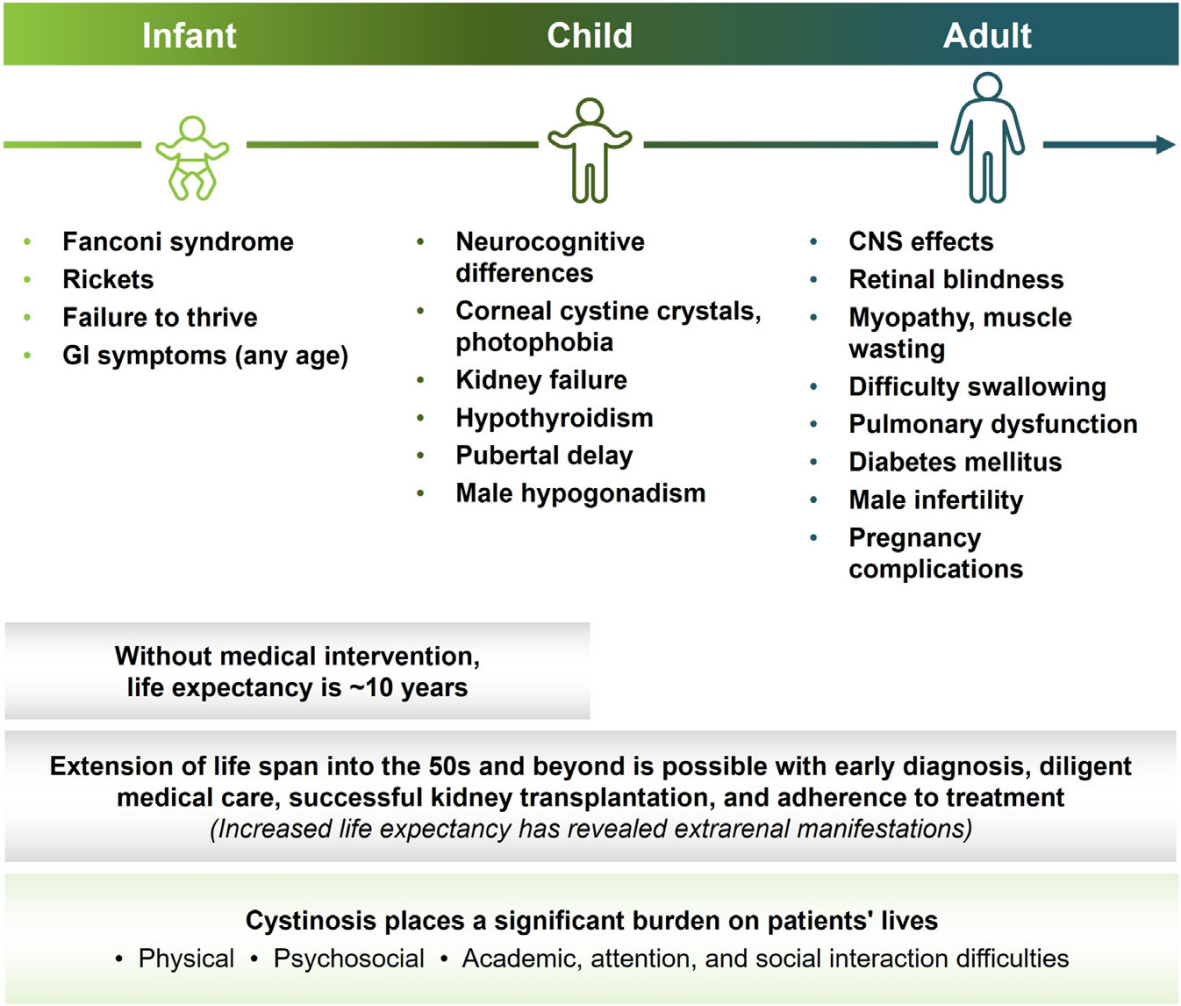
Progression of nephropathic cystinosis in untreated and undertreated patients. Each symptom is listed with the age of onset where it most commonly begins. As patients age, symptoms continue advancing, with additional organ systems involved.^2^^,^^3^ CNS, central nervous system; GI, gastrointestinal.

Infantile nephropathic cystinosis, the most common and severe form of the disease, is an ultrarare autosomal recessive lysosomal storage disorder caused by variants in *CTNS* and characterized by progressive cystine accumulation and multiorgan damage.^1^ It was once considered a fatal childhood disease; however, kidney transplantation, earlier diagnosis, and cysteamine therapy have transformed its course.^1^^,^^2^ As life expectancy has increased beyond the age of 50s, an extrarenal phenotype has been revealed, with impacts to nearly all body systems (Figure 1).^2^^,^^3^ Here, we describe the complicated clinical course of an adult with cystinosis and multiple comorbidities, to enrich the understanding of the disease and identify opportunities to support patients in adulthood.

## Case Presentation

### Diagnosis and Initial Management

The patient is a 36-year-old female who was diagnosed with infantile nephropathic cystinosis at the age of 5 years after proteinuria was discovered during a routine well-child visit. Diagnosis was confirmed via slit-lamp eye examination and kidney biopsy; genetic testing later identified 2 pathogenic *CTNS* variants (Table 1). Treatment with oral immediate-release cysteamine bitartrate (Cystagon; Viatris Inc, Canonsburg, PA) commenced upon diagnosis, and cysteamine eye drops were started at the age of 14 years through a clinical trial.

**Table 1:** Clinical events, evaluations, and treatment history

Age at Diagnosis, yr	Complication	Initial assessment and routine evaluation	Treatment
5	Proteinuria, photophobia	Slit-lamp eye exam (cystine crystals); kidney biopsy (cystine crystals); genetic testing (57 kb deletion and c.971-12G>A *CTNS* variants)	Oral cysteamine, cysteamine eye drops (started at age of 14 yr)
9	Hypothyroidism	Thyroid panel	Levothyroxine
11	GERD	NA	Omeprazole, probiotic
	Dysphagia	Swallowing study (normal); repeated at ages of 21 yrs (normal), 27 yrs (mild hypopharyngeal stasis), 30 yrs (normal), and 33 yrs (normal)	Speech therapy
12	Kidney failure	NA	Kidney transplant; prednisone, tacrolimus, calcium, magnesium, vitamin D, ferrous sulfate
	Hypertension	BP monitoring	Atenolol
	PTLD (B-cell lymphoma)	NA	Chemotherapy
19	Migraine	NA	Sumatriptan
	Chronic pain	NA	Desvenlafaxine
	Chronic fatigue syndrome	NA	NA
20	Low back pain	MRI of lumbar spine (normal)	NA
	Gallstones, biliary colic	NA	Cholecystectomy
21	Pain in chest wall	Echocardiogram (mild tricuspid insufficiency); follow-up chest CT at 26 yrs (patchy ground glass attenuation in both lungs; infiltrate cannot be ruled out), exercise stress test at 26 yrs (exercise capacity below average; test terminated early due to fatigue), chest radiograph at 27 yrs (normal), chest CT at 28 yrs (normal), echocardiogram at 31 yrs (mild tricuspid insufficiency), echocardiogram at 35 yrs (normal)	NA
	Frequent headaches	Head CT (normal); follow-up brain MRI at 26 yrs (5–6 tiny punctate 3–5 mm foci of signal abnormality within the subcortical deep white matter of both cerebral hemispheres, including frontal and parietal lobes bilaterally), head CT at 28 yrs (normal), brain MRI at 29 yrs (5–6 punctate white matter abnormalities; new T1 bright dentate nuclei and basal ganglia), head CT at 35 yrs (normal)	NA
24	Menorrhagia	Abdominal CT (normal)	NA
	Intestinal dysmotility	NA	Probiotic
	Diverticulosis	NA	Probiotic
	Chronic nausea	NA	Ondansetron (discontinued, not effective)
	Dyspepsia	NA	Omeprazole
	Impaired night vision	Eye exam	Prescription glasses
	Depression/anxiety	Psychological assessment	Desvenlafaxine, lorazepam; therapy
26	Fibromyalgia	NA	NA
	Gastroparesis, abdominal pain, bloating	Gastric emptying studies (delayed gastric emptying at 4 h); abdominal CT (mild inflammatory changes in LLQ consistent with epiploic appendage); transabdominal and endovaginal US (small amount of fluid in pelvic cul-de-sac); follow-up abdominal CT at 27 yrs (2 cystic lesions of the lateral R native kidney, stable mild transplant caliectasis, isolated sigmoid diverticulum, tiny fat-containing paraumbilical hernia, 2.7 cm functional R ovarian cyst), abdominal radiograph at 28 yrs (normal), abdominal CT at 30 yrs (mild wall thickening and possible inflammation of the bladder, cysts on both ovaries), transabdominal and endovaginal US at 33 yrs (normal), gastric emptying studies at 35 yrs (moderate delay in gastric emptying)	NA
	Myopathy, bilateral hand numbness, difficulty with squeezing hands, opening jars	Hand and wrist radiographs (remote fracture of the left ulnar styloid)	NA
27	Lyme disease	Brain MRI	Antibiotic protocol
28	Abdominal pain, nausea	Abdominal radiograph (normal)	NA
	Globus sensation	Head and neck US (normal)	NA
29	Right shoulder pain	Shoulder radiograph and MRI (normal)	NA
30	Type 2 diabetes mellitus	Laboratory testing	Metformin, glipizide
31	Kyphosis of cervical spine	Radiograph (shallow kyphosis); MRI (no significant findings)	NA

BP, blood pressure; CT, computed tomography; GERD, gastroesophageal reflux disease; LLQ, lower left quadrant; MRI, magnetic resonance imaging; NA, not applicable; PTLD, posttransplant lymphoproliferative disorder; R, right; US, ultrasound.

### Medical History

The patient experienced gastrointestinal challenges throughout childhood and began reporting mild dysphagia at the age of 11 years. Subsequent swallowing study results were normal. She underwent kidney transplantation at the age of 12 years and later developed hypertension and posttransplant lymphoproliferative disorder (B-cell lymphoma) secondary to immunosuppressive therapy. She experienced various extrarenal complications and underwent additional screening in adulthood, including echocardiograms, brain magnetic resonance imaging, hand and wrist radiographs, gastric emptying or gut transit time studies, and swallowing tests (Table 1).

### Cysteamine History

It is unclear how well-controlled this patient’s white blood cell (WBC) cystine levels were before the age of 17 years or if there were periods of nonadherence, because this information along with cysteamine history is not available to her current clinic (Table S1). She was prescribed immediate-release cysteamine until the age of 29 years; however, she never tolerated the full dosage due to nausea and abdominal pain. She underwent routine WBC cystine level monitoring, which indicated adequate short-term cystine depletion. However, many of these measurements were not true trough levels (taken less than 6 hours after last immediate-release cysteamine dose^4^), making it difficult to assess disease control. At the age of 29 years, the patient switched to delayed-release cysteamine bitartrate (Procysbi; Amgen Inc, Thousand Oaks, CA) to assess the potential effect of every-12-hour dosing on gastrointestinal symptoms. Delayed-release cysteamine was initiated at a low dosage, with the goal of slowly titrating to an appropriate maintenance dosage; gastrointestinal distress continued during the titration process. The patient found that consuming crackers or very low-fat bread with cysteamine minimized her symptoms.

The patient is currently taking 75% of her goal delayed-release cysteamine dosage. Although her last WBC (granulocytes) cystine level was elevated at 5.73 (goal: <1.9 nmol ½ cystine/mg protein), it was drawn 36 hours after the last delayed-release cysteamine dose and was likely not representative of a true trough value.^4^ In addition to oral and ophthalmic cysteamine, she takes levothyroxine, atenolol, prednisone, tacrolimus, sumatriptan, probiotic, omeprazole, desvenlafaxine, lorazepam, metformin, glipizide, and various supplements.

### Transition to Adult Care and Multidisciplinary Care

The patient began transitioning from pediatric to adult care at the age of 20 years while attending college out-of-state. She transferred to a metabolic genetics center of excellence for lysosomal storage disorders at the age of 26 years after difficulty finding an adult nephrologist willing to provide comprehensive cystinosis care. She is routinely followed-up with by specialists in genetics, nephrology, internal medicine, oncology, ophthalmology, psychiatry, and dermatology. Additional specialist referrals are handled as needed. The patient was previously evaluated for possible autoimmune diseases by a gastroenterologist, a rheumatologist, and a neurologist.

## Results

**Figure 2:**
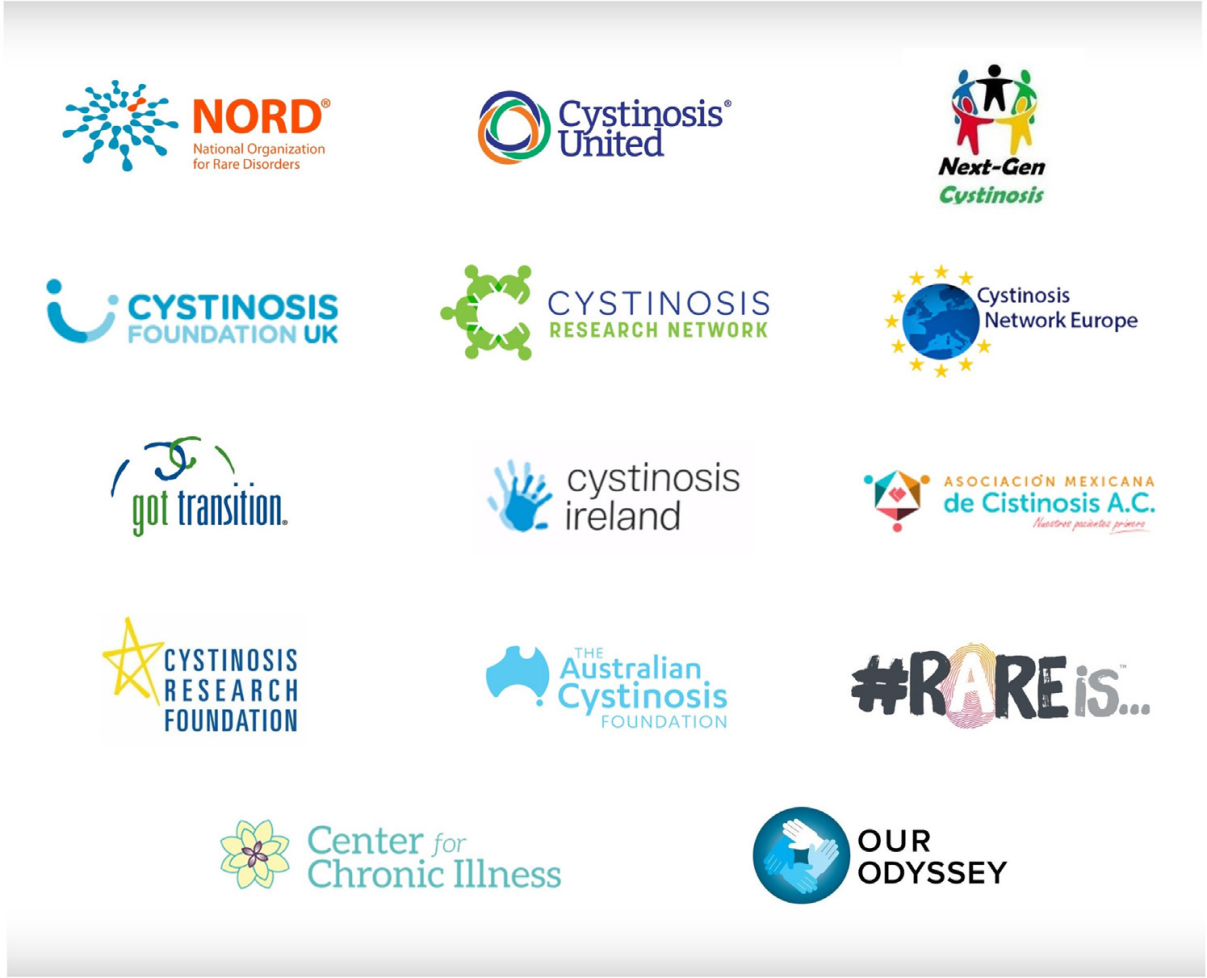
Resources to support the care of adults with cystinosis. Select education, patient advocacy, and peer support resources for adults with cystinosis and other rare diseases.

Due to her physical limitations, the patient is unable to work in a traditional job and relies on disability benefits. Although she maintains her independence and lives alone, her parents are involved in her care and offer continued support. She also has a life partner, who often attends medical appointments. Despite the progressive nature of her multisystemic complications, the patient lives a full life and uses her experiences and skills obtained from postsecondary education to bring awareness to the rare disease community (Figure 2). She is an active volunteer and is highly involved in a patient advocacy group for cystinosis.

## Discussion

As seen in this case report, the physical manifestations of cystinosis, as well as its treatments, can profoundly impact patients’ quality of life, professional development, and relationships.^3^^,^^5^ Although this patient has received many diagnoses, her subjective experience more fully captures her true disease severity. She has trouble with ambulation; pain and limited dexterity in her hands; swallowing difficulties; and visual impairment that makes driving difficult, particularly at night. Most notably, she experiences intense generalized pain and fatigue, which have limited her professional and personal aspirations. Although she is well-educated, she is unable to work in a conventional job due to lack of endurance, inability to meet expectations, and the need for frequent sick days.

This patient has undergone extensive testing for her symptoms, which has shown largely normal results. Standard assessments for common symptoms of cystinosis, such as swallowing difficulties, are often not sensitive enough to capture patient-reported abnormalities.^6^ In addition, certain extrarenal complications, such as gastrointestinal challenges, are multifactorial, making it difficult to determine and address the underlying etiology.^7^

Oral cysteamine is the only disease-modifying systemic treatment available for cystinosis. It has been shown to delay the time to kidney transplant and limit the extent of extrarenal complications with early and sustained use.^2^^,^^8^^,^^9^^,^S1–S3 The importance of prompt cysteamine initiation was confirmed in a natural history study of 86 adults with cystinosis. Patients who began treatment after 5 years of age had a significantly faster progression to kidney failure and greater incidence of other complications, including hypothyroidism, diabetes, neuromuscular challenges, and death, compared to those who started cysteamine earlier.^8^ More recent publications emphasize the need for even earlier diagnosis and treatment initiation before the onset of glomerular damage to promote optimal patient outcomes.^9^^,^S1

At the age of 5 years, the patient’s cystinosis diagnosis was delayed, which may have contributed to worsened overall outcomes. It is also unclear how well-controlled her disease was in childhood. In adulthood, her WBC cystine levels appear to reflect appropriate short-term cystine depletion, although she continues to be unable to tolerate her target cysteamine dosage. Patients with cystinosis require lifelong cysteamine treatment; those who are unable to tolerate the goal dosage should take their maximum tolerated dosage once it has been established. This provides some disease mitigation but may not be optimal.

Cystinosis places a large burden on patients’ lives, with impacts on physical functioning, relationships, autonomy, and social status.^5^ In cases like the one described, patients may be blamed for lack of adherence due to their inability to tolerate cysteamine or other factors. To support this patient, the genetics clinic explored alternative dosing techniques to reduce adverse effects, provided and reinforced education on appropriate timing of WBC cystine level testing, and continued to encourage medication adherence while promoting the concept of progress over perfection. In addition, the clinic determined the patient’s maximum tolerated cysteamine dosage and slowly titrated to this individualized goal. Clinicians and patient advocacy groups must be willing to navigate the new and evolving landscape of cystinosis care, with the goals of better understanding what it means to live with cystinosis as an adult, and to develop collaborative strategies to address the unmet needs in this patient population (Table 2). Considering that the nephrologist must be highly focused on the specialists’ responsibilities, the broader scope of the patient’s multisystem disease may be overlooked unless there is a primary care provider, or clinical geneticist, who takes responsibility for the comprehensive coordination of the patient’s complex medical care.

**Table 2:** Teaching points

Cystinosis in adulthood
• Physical health, psychosocial well-being, and overall quality of life are significantly affected for adults with cystinosis due to the multisystemic impacts of the disease and its treatments.
• Patients with cystinosis require lifelong cysteamine treatment; those who are unable to tolerate the goal dosage should take their maximum tolerated dosage once it has been established.
• Throughout life, in childhood and adulthood, WBC cystine levels should be monitored regularly to assess “cystine depletion” and the need for cysteamine dosing adjustments; levels must be evaluated against the standardized last dose of IR cysteamine^a^ (6 h) or DR cysteamine^b^ (12 h) dose to determine the true trough level of cystine.

DR, delayed-release; IR, immediate-release; WBC, white blood cell.

a Trough level should be obtained approximately 6 h after the last dose of IR cysteamine, but before the next dose.^4^

b Trough levels should be obtained 12 to 12.5 h after the last DR cysteamine dose, but before the next dose.^4^

## Disclosure

All the authors declared no conflicting interests.

## Patient Consent

The authors declare that they have obtained consent from the patient discussed in this case report.

## Supplementary Material

Supplementary File (PDF) **Table S1.** Cysteamine and WBC cystine level history.**Supplementary References.**

## Footnotes

Supplementary File (PDF)
**Table S1.** Cysteamine and WBC cystine level history
**Supplementary References.**
