# Supplementary material for: Addressing the Multisystemic Impacts of Nephropathic Cystinosis in an Adult
Source: Kidney Int Rep. 2025 Mar 4;10(3 Suppl):S789–93. doi: 10.1016/j.ekir.2024.10.039 (PMC11935152; doi:10.1016/j.ekir.2024.10.039)
Supplement: Supplementary File (PDF) — Table S1. Cysteamine and WBC cystine level history.Supplementary References. [file mmc1.pdf]

SUPPLEMENTARY TABLE S1. Cysteamine and WBC cystine level history.

Age,[a] y	Cysteamine Formulation	Total Daily Cysteamine Dose Prescribed, mg[b]	Total Daily Cysteamine Dose Taken, mg[b]	WBC Cystine Level,[c] nmol ½ cystine/mg protein	Time Since Last Dose, h
17.8	IR	3600	1200-1800	0.20 (ML)	3.7
18.2	IR	3600	1200-1800	0.20 (ML)	NA
18.6	IR	3600	1200-1800	0.10 (ML)	NA
19.0	IR	3600	1200-1800	0.10 (ML)	2.3
19.8	IR	3600	1200-1800	0.09 (ML)	3.9
20.0	IR	3600	1200-1800	0.08 (ML)	4.1
20.8	IR	3600	1200-1800	0.21 (ML)	2.3
21.3	IR	3600	1200-1800	0.05 (ML)	2.4
22.3	IR	3600	1200-1800	0.18 (ML)	2.4
26.8	IR	3600	1200-1800	0.06 (ML)	4.7
28.5	IR	3600	1200-1800	0.74 (ML)	NA
29.5	DR	150	150	1.84 (G)	12.6
29.6	DR	NA	NA	1.10 (G)	12
29.9	DR	2000	1500	1.39 (G)	NA
30.5	DR	NA	NA	1.09 (G)	12
31.5	DR	NA	NA	2.26 (G)	11
32.6	DR	NA	NA	0.87 (G)	9.7
36.8	DR	2000	1125	5.73	36

[a]Patient records unavailable for cysteamine use and WBC cystine levels before age 17 years.
[b]IR cysteamine dosed every 6 hours; DR cysteamine dosed every 12 hours.
[c]Mixed leukocytes cystine level goal: below 1.0 nmol ½ cystine/mg protein; granulocytes cystine level goal: below 1.9 nmol ½ cystine/mg protein.[S4]
DR, delayed-release; G, granulocytes; IR, immediate-release; ML, mixed leukocytes; NA, not available; WBC, white blood cell.

SUPPLEMENTARY REFERENCES

S1. Hohenfellner K, Nieβl C, Haffner D, et al. Beneficial effects of starting oral cysteamine treatment in the first 2 months of life on glomerular and tubular kidney function in infantile nephropathic cystinosis. *Mol Genet Metab*. 2022;136(4):282-288. https://doi.org/10.1016/j.ymgme.2022.06.009

S2. Nesterova G, Williams C, Bernardini I, et al. Cystinosis: renal glomerular and renal tubular function in relation to compliance with cystine-depleting therapy. *Pediatr Nephrol*. 2015;30(6):945-951. https://doi.org/10.1007/s00467-014-3018-x

S3. Gahl WA, Balog JZ, Kleta R. Nephropathic cystinosis in adults: natural history and effects of oral cysteamine therapy. *Ann Intern Med*. 2007;147(4):242-250. https://doi.org/10.7326/0003-4819-147-4-200708210-00006

S4. Gertsman I, Johnson WS, Nishikawa C, et al. Diagnosis and monitoring of cystinosis using immunomagnetically purified granulocytes. *Clin Chem.* 2016;62(5):766-772. https://doi.org/10.1373/clinchem.2015.252494
